# Excerpts from the Medical Press

**Published:** 1860-02

**Authors:** 


### EXCERPTS FROM THE MEDICAL PRESS.

BY J. A. A.

The medical journals of the country are exhibiting a very laudable activity in collating the more recent and reliable experiments and observations, and the results of *experience* in practice, to the prominent exclusion of merely speculative essays.

In the experimental department, the indefatigable M. Bernard still illustrates both industry and sagacity, and attracts general notice. From his recent experiments upon the "Quantity of Oxygen in the venous blood of glandular organs when in activity and repose," we extract the following: (translated by Dr. E. Warren, *Med. Jour., N. C.*)

"These experiments demonstrate that the red venous blood of the kidney (and it is presumable that the same is true of the other glandular bloods) differs from ordinary blood, in that it is not *deoxydized.* Thus is our original hypothesis verified, in as much as this blood preserves the characteristics of arterial. However, if this is true for the proportions of oxygen discovered therein, the proposition is not absolutely exact. In fact, the red glandular venous blood contains much less fibrine than the arterial; there is less water in it, because that of the secretions has been substracted; and also, it is more susceptible of alteration (decomposition), that is to say, it becomes spontaneously black much more rapidly when exposed to the atmosphere.

However this may be, in observing for the moment the veritable object of our research. *i. e.*, what concerns the proportion of oxygen in glandular venous blood, we perceive this remarkable fact—it is whilst performing their functions at the moment of secretion, that the glands permit the blood to circulate without deoxydizing it, and it is whilst they are not acting, and when no product is expelled, that the blood which escapes is dark, deprived in a great measure of its oxygen, and charged with carbonic acid.

Here again appears the antagonism between the glandular and muscular systems, to which I have often referred. In the muscles, the blackness and deoxydation of the blood passing through them are in direct proportion to the energy of their contractions. In the glands the blood is more red, and less deoxydized, in direct proportion to the activity with which the secretory function is performed. Should we not regard this antagonism in the phenomena thus made apparent, as indicating a *radical* difference both in the modes of nutrition, and the functions of glands and muscles? In a word, may we not affirm that the muscles consume oxygen in direct ratio to their activity of function—as is directly opposite with the glands? Or should we rather, in view of this singular phenomenon, conceive doubts respecting the truth of our conclusions in regard to the functional state of the glands?

I incline to the latter opinion; and I think that these researches will lead to another interpretation of that which has heretofore been esteemed the states of activity and of repose of glandular organs, and will induce us to distinguish a state of chemical activity, and another purely mechanical. I could easily adduce several arguments in support of this opinion; but I will conclude with the simple facts above enumerated, limiting myself to a simple eunciation of this obscure proposition, which will serve as a point of departure for future explorations."

---

Influence of Calomel on the Liver.—Dr. George Scott, of Southampton, England, as the result of four experiments upon dogs, concludes that so far from the biliary secretion being increased by the administration of calomel in large doses, it is absolutely diminished in quantity. It will occur to the careful student, that even admitting the results of the particular experiments, no practical inference is to be derived from them, except perhaps that, as as has been long claimed by practioners in tropical climates, *large* doses of calomel are sedative rather than stimulant to the biliary apparatus.

The experiments are open to the following objections:—First, the effect of calomel in small and repeated doses is not noted; Second, the relative date of tying the common duct, is not given; Third, positive evidence in the possession of every practioner, that in the human subject, bile in large quantities can be educed by administration of the mercurial, almost at will. Positive experience must still rule, rather than questionable experiment.

---

Opium in Poisoning by Stramonium, etc.—Dr. T. L. Maddin, in the *Nashville Med. Record*, reports a case of poisoning by the seeds of Datura Stramonium successfully treated by Laudanum. Removal of such portions of the poison as remained in the intestinal canal, being first provided for by emesis and catharsis. The controlling power of the opiate over the constitutional disorders was distinct. Dr. M. urges the opium as a counter poison also, to belladonna, and similarly acting agents, considering "the pupil in all cases as a reliable index as to how far with safety we may proceed in its use."—The constitutional effects of the opiates, he deems likely to be relieved by belladonna, etc., under similar physiological reasons.

---

The Vomiting of Pregnancy.—In passing upon applications to the uterus in these cases, I would take the liberty of calling attention to the application of the tincture of benzoin and chloric ether, which I have been using with as good an amount of success as any agent I have employed, and it has the advantage of being very simple. I would particularly recommend it where there is much neuralgic pain and excessive leucorrhœal secretion. I have nothing so beneficial in these last named accompaniments as this preparation. By adding a few grains of acetate of morphia to this, it will also be found a very efficient remedy in painful menstruation, and will seldom fail, in the practioners hands, of giving ease and comfort to the female during this her much dreaded period, if applied just before or at the commencement of menstruation. It should be painted upon the os and cervix, once in three or four days; and may be continued throughout the whole period of pregnancy without any unpleasant effects. My formula for this preparation:—℞ Tinct. benzonii c., ʒ ii.; chloric ether ʒ i.; acet. morphia, grs, ii. M. These applications should not only be made to the mouth of the womb, but should extend to the neck, but if we wish to gain a favorable result from them. If the silver be used, it cannot be applied too lightly; a very slight pencilling is all that is required. If applied so as to produce sloughing and discharges, it will fail of accomplishing our desires. Are not the good effects of these applications to be accounted for, from the fact that they form a protecting coat over the congested and abraded uterine mouth and neck, that may take place in the impregnated uterus, thereby allaying the irritation of the external uterine nerves and vessels, by equal pressure and protection from all external influences of vagina and other sources? I should be happy to hear the result of the use of these preparations in the practice of other medical gentlemen.

In addition to the above, I have used injections of Ferri alumenis, ʒ i.; inf. opii, ℥ ii.; aquæ dist. ℥ viii. M.—with some benefit. These can be used where it would not be expedient to employ the speculum. I sometimes substitute iodide of zinc for the alum. This is more applicable to these cases where there is slight spasmodic action in this organ, or in the neck of the bladder. I use five grains of zinc to the ounce.—*Dr. J. H. Warren, in Boston Med. and. Sur. Jour.*

It appears quite possible that all the good effect assumed by Dr. Warren to result from the local application he suggests would be equally gained by anodyne injections *per rectum.*—It is scarcely probable that there is in these cases such a local condition of the *os* or *cervix uteri*, as to require use of the speculum every three or four days, or even more rarely. The speculum is abused quite enough already.

---

Remittent Fever in St. Louis.—The Medical Society of St. Louis, (reported in the St. Louis *Med. and Sur. Jour.* for November.) state that the several forms of malarial fever have been unusually abundant in that city the past season. At the O'Fallon Dispensary, 138 cases were prescribed for in a single afternoon. The type seemed protean. Quina and calomel were found as useful as ever, and largely relied upon.—Dr. Boisliniere suggested dry cupping over the epigastrium, as very useful in allaying initation and vomiting. Dr. Pallen recommended mixing the quina with champagne, to quiet the stomach and prevent vomiting. He has found this practice very useful in many cases.

---

Puerperal Convulsions.—Dr. M. M. Pallen, in the same journal, suggests "that the heart during pregnancy enlarged as well as the uterus, and by its increased size propelled more blood with greater force towards the brain than at any other time, and thus created a greater tendency to congestion of the brain and consequent convulsions."

It is sincerely to be hoped subsequent investigations may not corroborate this opinion, for if they do so, there will be great temptation to employ bloodletting and other depletives, beyond even their present unwarrantable application in this form of disease. Cases may recover after their employment, and we think such recoveries ought, from their extraordinary character, to be duly heralded. But, meantime, physiology, and enlightened pathology point to the nervous system for the seat of the disease, and to the vascular system only for symptoms and results. Remedies for puerperal convulsions must be sought among the means capable of controlling irregular *reflex* actions. Stimulants and other agencies which powerfully impress the nervous centres, are quite as likely to prove useful, theoretically and practically, as depletives, or perhaps even sedatives.

---

Bibron's Antidotes for Snake Bites.—This compound still continues to attrack attention, and several cases of its complete success have been reported. The formula has been previously published in this Journal. The idea was unquestionably suggested to the putative inventor, by Prof. Brainard's well known experiments before the Committee of the Academy of Medicine, at Paris, several years since.

---

Pseudo Membranous Laryngites.—Dr. Estep, in the Belmont *Med. Journal*, remarks of this affection:

"My anchor of hope for almost unfailing success, is in opium and calomel; the former in the form of Dover's powder in from 3 to 6 gr. doses, owing to the age of the child, combining it with from 1½ to 3 grs. of calomel, repeated every two hours, until the disease is declining, then the dose is diminished in size and frequency, but continued until a cure is completed; the condition of the bowels of course to be attended to."

Dr. E. attributes most credit to the opium. He subjoins counter irritation to the throat, warm baths, etc., as beneficial. "With this treatment," he observes, "croup will be disarmed of its dangers, and not one in fifty will die if the treatment is adopted before the third stage, or stage of collapse."

---

TREATMENT OF SCARLATINA BY IODINE.—W. Reeves, M. R. C. S., in the London *Lancet*, recommends in all cases of scarlatina as soon as diagnosticated: ℞ Potassii Iodid. ℨ i.; Iodine, gr. ii; Potassæ Chlorat. ℨ i.; Potassæ Nitrat. ℨ iss.; Aquæ, ℥ viii. M. From a teaspoonful to a tablespoonful every four hours, according to the age of the patient. He adds to this, local application of the iodine to the throat internally by means of a feather, and externally in the form of ointment over the glands. He claims that "there are no other remedies in this disease at all to compare with iodine, thus employed.

---

INGROWING NAIL.—The Nashville *Journal* recommends in this annoying difficulty, scraping off the middle of the nail with a bit of glass until it is as thin as possible. "It is of no use to pare the edges of the nail, for this merely increases the torture; but as soon as the middle has been thinned, it yields to the upward pressure of the skin on its side edges, readily bends and offers no more resistance. Then the proud flesh drops down and the sore heals."

We can endorse this very simple and painless process, from frequent experience in its use for a dozen years past.

---

HYDROCELE.—Mr. Pollock, in the London *Lancet*, suggests the use of a silver wire in place of the ordinary seton, or other remedial measures. He first evacuates the fluids and then allows the wire to remain until sufficient irritation is produced to cause adhesion or obliteration of the morbid tendency to effusion.

We venture to suggest from experience with the single linen thread we have often employed, that it is better to allow the fluid to drain off gradually by the thread, or by the side of the wire. Much of it finds its way into the investing areola tissue, and by the pressure produced adds materially to the chances of salutary adhesion. We have thus succeeded in effecting cures of this troublesome difficulty in several cases which had previously resisted injections of various kinds.

---

Milk Sickness.—Dr Nagle in the Nashville *Journal*, (Oct.) asserts that he has discovered the cause of this disease to be the ingestion by cattle of an ergotized cereal grass, found upon swamps or prairies. By exhibition of this fungus growth to animals, he has been able to produce the disease at will.—Salt culture destroys this tendency to fungus growth in the grasses. The main object of treatment is tonic and stimulant, and particularly to keep the bowels open. For these objects he relies upon calomel, or blue pill, and quina in large doses. Excessive constipation is met by clysters of cold water with a little salt, and renewed from time to time until feculent dejections occur. To this is sometimes conjoined judicious doses of elaterin. Dr. Nagle regards corn meal as an absolute specific in the treatment of the usually tedious convalescence, as much so as hydrated peroxide of iron for arsenic. "Not only in man is it a specific, but in cattle the cure is certain. Feed either party with it in plenty, give them all they can possibly eat, give it as mush and milk, as corn bread, as raw corn meal or any other manner you can, but give corn to cure milk sickness."

Typhoid Fever.—Dr. Bowling, in the same journal, in a rambling but genial style of comment upon the treatment of typhoid fever, taking a letter from Dr. Cogley as his text, calls upon the profession to return to the famous Nathan Smith, or "buttermilk treament," simply conjoining turpentine as a specific remedy. Not as Prof. Wood suggests in a particular stage of the disease, but in every stage, and persistently.—The Doctor enthusiastically remarks: "It is hardly saying too much that NATHAN SMITH AND TURPENTINE WILL CURE TYPHOID FEVER," He ignores all other treatment. Buttermilk *ad libitum*, and Sp. Terebinthi, five drops every three hours! Only this and nothing more. Surely here is a climax of simplicity—a consummation devoutly to be wished—if true. But in another page of the same number, we find a correspondent lauding the peculiar treatment adopted by Doctor Thompson, which he assumes is destined to supplant every other, as soon as it is generally known. *Ecce signum*—dry cups and powerful rubefacients to the spine, potent nerve stimulants internally, and then quina in ten grain doses. So it appears that Dr. Bowling has not yet converted his own state to his views. Perhaps on the whole, he is nearer right, but *in media tutissimus ibis*.

---

STRYCHNIA IN CHRONIC INTERMITTENTS.—In the New Orleans *Medical News and Hospital Gazette*, Dr. Harrison, of Arkansas, has an article upon the use of strychnia in chronic intermittents.

The following is his formula:

| | | | |
|---|---|---|---|
| " ℞.— | Strychnia, | gr. iss. | |
| | Sulph. Quinine, | gr. xv. | |
| | Capsicum, | gr. vj. | |
| | Brandy, | ℥ iv. | M. |

"Of this mixture, I direct one teaspoonful (for an adult) every hour, for six or seven hours preceding the expected paroxysm; at the end of this time I require the patient to take a cup of warm sage tea, and go to bed, (if he is not already there,) and remain until the paroxysmal hours pass. This course is to be repeated on the next 'chill day,' after which a teaspoonful of the medicine is to be taken two or three times per day, until the four ounces are exhausted."

With Dr. H. F. Campbell's views of the nature of this disease, and its relation to the nervous system, the philosophy of this treatment at once becomes apparent. This, however, is not altogether a new treatment. Dr. Brainard, of Chicago, recommended strichnine, in an eighth-of-a-grain dose, three times a day, in similar cases, more than twelve years ago.—(*See Indiana Med. Jour., July*, 1847.)

We are confident that the remedial powers of strichnia are not yet fully brought out. So far as we know, we were the first to use and recommend it, in *sciatica* and chronic rheumatism; and we have seen cases of dyspepsia and chronic costiveness yield to it like a charm.—*N. Y. Med. Monthly.*

---

Direct injection of Nitrate of Silver, etc., to the Stomach, in severe Dyspepsia and Chronic Inflammation.—Dr. Alexander Fleming, senior physician to the Queen's Hospital, Birmingham, after alluding to the ordinary mode of exhibiting remedies, proceeds:

"But this method of exhibiting the medicine is not equal to the cure of some of the severer forms of dyspepsia and chronic gastritis; and in these, I have, for the last four years, endeavored to act more generally and efficiently on the mucous surface by injecting the solution into the stomach. I employ a strong brass syringe and flexible tube—one-eighth of an inch in bore—the gastric end of which has a number of holes so directed that the fluid is thrown in a circular shower outwards and upwards on the walls of the stomach. The injection is made by dissolving from one to four grains of the nitrate in three ounces of distilled water. The operation is for the most part managed easily. Sometimes it causes nausea and retching—oftener not. It excites at first an enduring and grateful sense of coolness in the stomach; and subsequently there are felt pricking and sharp painful sensations, but of a different nature from the pains of the disease. Sometimes one injection is enough; but I have more frequently had to repeat it two, three or more times.

During the employment of the injections, the patient takes three times a-day and before food a little morphia or chloric ether, or Indian hemp, in plain or cinnamon water. He is confined to small and frequent meals of milk; and as he gets better this is thickened with arrow-root or tapioca, and he is gradually introduced to a nourishing and easily digestible diet. Counter-irritation to the epigastrium, of bismuth, oxide of silver, gentle tonics, &c., are employed when indicated.

Of the thorough efficiency of this mode of acting on the mucous surface of the stomach, and of its power in promoting the cure, my experience, so far as it goes, is very decided.—Although it is now four years since I tried injection, I have not used it in more than ten cases. I have always, in the first instance, employed the simpler method already described, and resorted to an injection only as a last resource: but its greater efficiency would, I feel certain, justify its employment in many of the less severe cases, and give more thorough and speedy cures. It is not my purpose at present, to consider the intimate nature of the mode of cure, or the manner in which the nitrate of silver substitutes healthy for diseased action in the inflamed gastric membrane. I must reserve that interesting question, and the detailed narrative of cases for another opportunity.

---

Adulterations of Food and Medicine.—The committee on this subject, of the American Pharmaceutical Association, in their last report, specially refer to the following as most frequent.

Colored Confectionery—Adulterated with emerald or Scheele's green, arsenite of copper.

Beer—with cocculus indicus and nux vomica.

Pickles and Bottled Fruit—with verdigris and sulphate of copper.

Custard Powders—with chromate of lead.

Tea and Snuff—with the same.

Cayenne and Curry Powder—with red oxide of lead.

Sugar Confectionery—with gamboge, orpiment, or sulpuret of arsenic, and chloride of copper.

Flour and Bread—with hydrated sulphate of lime, plaster of Paris and alum.

Vinegar—with sulphuric acid.

Sugar—with sand and plaster of Paris.

Milk—with chalk, sheep's brains, ground turmeric.

Arrow root—with ground rice.

Chocolate—with rice flour, potato starch, gum tragacanth, cinnabar, bals. Peru, red ox. mercury, red lead, carb. of lime, and the red ochres to bring up the color.

Mustard—with ground tumeric, to give a brilliant color.

Butter—with potato starch, mutton tallow, carbonate of lead and sugar of lead.

One of the members of your Committee, who is acquainted with a gentleman formerly in the drug grinding business in New York, has been kindly furnished by him with some formulas by which "pure and genuine drugs" were prepared when he was employed at the mill referred to:

Powdered Cape Aloes—Cape aloes dried, 100 ℔s.; ship biscuit, 100 ℔s.; curcuma q. s. to color.

Common Ginger—African ginger, 200 ℔s.; capsicum hulls, 25 ℔s.; biscuit, 100 ℔s.; curcuma q. s. to color.

Ipecac. powdered—Ipecac., 100lbs.; ship biscuit, 25 to 40 pounds.

Opium powdered—Turkey opium, 50 lbs.; Egyptian opium, 25 lbs.; biscuit, 40 lbs.

Gamboge powdered—Gamboge, 100 lbs.; tartrate of lime, 25 lbs.

Socotrine Aloes are pure bonaire, without adulteration.

Cream of Tartar is adulterated with from 10 to 65 per cent. of terra alba, or tartrate of lime, with about 3 per cent. tartaric acid.

Tartaric Acid powdered—Tartaric acid, 1000 lbs.; alum, from 10 to 35 per cent.

Scammony Aleppo, powdered—Virgin Scammony, 30 lbs.; cocoa beans, 80 lbs.; biscuit, 30 lbs.; lampblack, q. s. (sufficient quantity) to color.

Bird Pepper, powdered—Chilies, 1000 lbs.; rice, 800 to 1200 lbs.; curcuma and Venetian red to color.

Powdered Fenugreek—Fenugreek seeds, 1000 lbs.; biscuit 1000 lbs.; curcuma q. s. to color.

East India Rhubarb, powdered—East India rhubarb, 100 lbs.; English do., 60 lbs.

English Rhubarb, powdered—English rhubarb, 100 lbs.; biscuit, 30 lbs.; curcuma to color.

Turkey Rhubarb, powdered—East India rhubarb, Turkey rhubarb, equal parts.

The tartrate of lime referred to is more properly sulphate of lime with a small portion of tartrate. The ship biscuit is the hard and often worm-eaten cakes brought in by ships after a long voyage.—*Am. Jour. Pharmacy.*

---

Therapeutic Properties of Sarsaparilla.—Dr. M. Adam, in some interesting *Medical Notes from the Continent*, refers to some experiments by Prof. Bocker upon sarsaparilla, as yet unpublished. Dr. Bocker told Dr. Adam, "that after carefully performing ninety-eight experiments with this drug on healthy people, he found that contrary to all our usually received opinions on the subject, it possesses neither diuretic nor diaphoretic properties. Another series of twenty-six experiments, on the persons of uncured syphilitic patients, gave exactly the same results." Bocker also satisfied himself that sarza does not increase the efficacy of the agents, such as iod. potass., &c., which are usually given along with it; and that the good results obtained by the administration of this salt, dissolved in decoction of sarza, are in no degree attributable to any virtue in the solvent fluid. I told Dr. Bocker that I

remembered hearing Prof. Syme, many years ago, express his opinion on the utter uselessness of so expensive a drug as sarza, remarking, in his own quaint, forcible style, that he believed an 'infusion of hay' would be just as good, and a vast deal cheaper. He seemed amused, and said that he entirely agreed with Syme; that infusion of sarza had no greater effect on the system than so much common tea; and that we must regard it merely as a pleasant, but very expensive, vehicle for the administration of other medicines.—*Edinburgh Med. Journal.*

---

SUBMUCOUS INJECTION AS A CURE IN THE TOOTHACHE OF PREGNANCY.—Horatio R. Storer, M. D., in the Boston *Med. Journal*, strongly recommends this process, rather than the dangerous and uncertain one of extraction. He instances the following:

"CASE.—A. Z., aged 22, applied to me for treatment early in May last. Patient had suffered for several weeks from severe neuralgic pain throughout the left half of the upper jaw, at times lancinating in its character, at others more dull, but never wholly absent. The general health was decidedly affected, as evidenced by the state of the circulatory, digestive, and nervous systems. The teeth, on inspection, were all sound; there was no heat or swelling of the gums, no tenderness or increase of pain on pressing them.

"Anodynes, both local and general, refrigerants, emollient poultices and counter-irritants, were successively resorted to, without benefit. After much solicitation a tooth was extracted; the patient remained unrelieved. On the following day, no change for the better having occurred, ten drops of the Edinburgh solution of the bi-meconate of morphia were injected beneath the mucous membrane of the gum; the pain ceased instantaneously, and from that moment to the present, a period of nearly five months, there has been no return of the malady."

(*To be continued.*)

---

GENIUS.—M. Moreau asserts that all men of genius are men in different states of madness. Genius is a neurosis.

